# Intertumor heterogeneity in vascularity and invasiveness of artificial melanoma brain metastases

**DOI:** 10.1186/s13046-015-0264-0

**Published:** 2015-12-15

**Authors:** Trude G. Simonsen, Jon-Vidar Gaustad, Einar K. Rofstad

**Affiliations:** Group of Radiation Biology and Tumor Physiology, Department of Radiation Biology, Institute for Cancer Research, Oslo University Hospital, Oslo, Norway

**Keywords:** Melanoma, Brain metastasis, Angiogenesis, Vascular co-option, Invasiveness

## Abstract

**Background:**

Patients diagnosed with melanoma brain metastases have few treatment options and poor prognosis, and improved treatment strategies for these patients require detailed understanding of the underlying pathobiology. In this investigation we studied the vascularity and invasiveness of artificial brain metastases established from four human melanoma cell lines.

**Methods:**

A-07, D-12, R-18, and U-25 cells transfected with GFP were injected intracerebrally and intra-arterially in nude mice. Moribund mice were killed and autopsied, and the brain was evaluated by fluorescence imaging or by histological examination. Expression and secretion of factors involved in angiogenesis and invasion were assessed by quantitative PCR, ELISA, and immunohistochemistry.

**Results:**

The melanoma cells grew preferentially in the meninges and ventricles after intracerebral and intra-arterial injection. Intertumor heterogeneity in the aggressiveness of meningeal tumors reflected differences in angiogenic activity and expression of vascular endothelial growth factor A (VEGF-A) and interleukin 8 (IL-8). In contrast, growth and invasion of the brain parenchyma relied primarily on vascular co-option. The cell lines showed different patterns of invasion from meninges to the scull and from meninges to the brain parenchyma, and these differences were associated with differences in expression of the matrix metalloproteinases MMP-2 and MMP-9. Furthermore, the melanoma cells produced multiple brain lesions after intracerebral implantation by using the meningeal linings of the brain as transport routes.

**Conclusions:**

The melanoma cell lines showed different growth patterns in the brain, and these differences were associated with differences in expression of the angiogenic factors VEGF-A and IL-8 and the matrix metalloproteinases MMP-2 and MMP-9.

## Background

Malignant melanoma frequently metastasizes to the brain and is the third most common cause of brain metastases after lung and breast cancer [1]. Clinical studies show that 30–50 % of patients with advanced-stage melanoma are diagnosed with brain metastases during the course of their disease, and the incidence of brain involvement increases to 50–75 % in autopsy studies [2–5]. Current treatment options for brain metastases, including surgical resection, stereotactic radiosurgery, and whole-brain radiotherapy, are mostly palliative and show low efficacy in melanoma patients [5, 6]. Consequently, the prognosis of melanoma patients diagnosed with brain metastases is generally poor, and many studies report a median survival of less than 6 months after diagnosis [4, 7, 8].

Development of improved treatment strategies for patients with melanoma brain metastases requires detailed understanding of the underlying pathobiology. Most clinical data are derived from retrospective studies based on either diagnostic CT or MRI images or on histological analysis of autopsy specimens. Such studies have shown that multiple lesions and intratumoral hemorrhage are characteristic features of melanoma brain metastases and they have identified important negative prognostic factors, including multiple brain lesions, the presence of extracranial metastases, and meningeal involvement [4, 7–10]. Studies of patient biopsies and autopsy specimens have also suggested that diffuse infiltration and growth along brain microvessels are common growth patterns in melanoma brain metastases [11, 12].

Although clinical studies have provided valuable insight into the biology of melanoma brain metastases, preclinical studies are required to study biological properties at a detailed level and to elucidate underlying mechanisms. Melanoma brain metastases have been studied preclinically by using animal models that include spontaneous brain metastases after orthotopic implantation, direct intracerebral tumor cell implantation, intra-arterial injection of tumor cells into the heart or the internal carotid artery, and cranial window chambers [13–15]. Two different patterns of melanoma metastases after intracarotid artery injection have been identified. Thus, some melanoma cell lines produce lesions in the brain parenchyma whereas others produce lesions preferentially in the meninges and ventricles [16, 17]. This site-specificity has been associated with expression of the transforming growth factor-β2 in a murine melanoma model [18]. Data on the microscopic invasion patterns within and between different brain compartments are however sparse. Preclinical studies have also demonstrated that vascularization of melanoma lesions within the brain parenchyma occur by both angiogenesis, i.e. sprouting of new blood vessels from pre-existing vessels, and by vascular co-option, i.e. growth of melanoma cells along pre-existing vessels [15, 19–21]. Angiogenesis within the brain has been shown to depend on the vascular endothelial growth factor A (VEGF-A) in melanoma as well as lung, colon, and breast carcinoma models [19, 22, 23]. However, so far, most studies demonstrating vascular co-option in melanoma brain metastases have used melanoma cell lines with low expression of VEGF-A [15, 20].

The human melanoma cell lines A-07, D-12, R-18, and U-25 have been established and extensively studied in our laboratory [24], and they have been shown to differ substantially in angiogenic signature, angiogenic potential, and metastatic properties when inoculated orthotopically in nude mice [25, 26]. The angiogenic potential of these melanoma cell lines is associated with expression of VEGF-A and interleukin 8 (IL-8) [25]. Furthermore, the invasiveness of A-07 and D-12 cells in vitro has been shown to depend on the matrix metalloproteinases MMP-2 and MMP-9 [27]. MMP-2 and MMP-9 are proteolytic enzymes that degrade type IV collagen and other components of the extracellular matrix, and they have been associated with tumor cell invasion, metastasis, and angiogenesis [28].

The purpose of the study reported here was to use A-07, D-12, R-18, and U-25 cells as models to study intertumor heterogeneity in vascularity and invasiveness of melanoma brain metastases. Artificial brain metastases were established by intracerebral and intra-arterial injection of melanoma cells transfected with green fluorescent protein (GFP), and vascularization and invasion patterns in the brain parenchyma, ventricles, meninges, and scull bone were studied in detail by fluorescence imaging and histological analysis. We report that the melanoma cell lines showed highly different patterns of vascularization and invasion in the brain and, furthermore, these differences were associated with differences in expression of the angiogenic factors VEGF-A and IL-8 and the matrix metalloproteinases MMP-2 and MMP-9.

## Methods

### Mice

Adult (8–10 weeks of age) female BALB/c *nu/nu* mice were used as host animals. The mice were bred at our institute and maintained under specific pathogen-free conditions at a temperature of 22–24 °C and a humidity of 30–50 %. The animal experiments were approved by the Institutional Committee on Research Animal Care and were performed according to the Interdisciplinary Principles and Guidelines for the Use of Animals in Research, Marketing, and Education (New York Academy of Sciences, New York, NY).

### Cell lines

A-07, D-12, R-18, and U-25 human melanoma cells [24] were obtained from our frozen stock and maintained as monolayers in RPMI 1640 (25 mM HEPES and L-glutamine) supplemented with 13 % bovine calf serum, 250 μg/ml penicillin, and 50 μg/ml streptomycin. The cultures were incubated at 37 °C in a humidified atmosphere of 5 % CO_2_ in air and subcultured twice a week. For in vivo experiments, cells constitutively transfected with GFP were used [29]. Cells were harvested from exponentially growing cultures and re-suspended in Ca^2+^-free and Mg^2+^-free Hanks’ balanced salt solution (HBSS) before injection into animals.

### Anesthesia

Intracerebral and intra-arterial injection of tumor cells were carried out with anesthetized mice. Fentanyl citrate (Janssen Pharmaceutica, Beerse, Belgium), fluanisone (Janssen Pharmaceutica), and midazolam (Hoffmann-La Roche, Basel, Switzerland) were administered intraperitoneally (i.p.) in doses of 0.63 mg/kg, 20 mg/kg, and 10 mg/kg, respectively. The body core temperature of the mice was maintained at 37–38 °C by using a heating pad.

### Intracerebral tumor cell injection

The mice were fixed in a stereotactic apparatus (Model 900; Kopf Instruments, Tujunga, CA) for injection of tumor cells into the right cerebral hemisphere. The injection point was 2 mm anterior to the coronal and 1 mm lateral to the sagittal suture lines. A 100 μl Hamilton syringe with a 26-gauge needle was used to inject 6 μl of cell suspension at a depth of ~3 mm below the scull. To minimize tumor cell reflux, the cells were injected slowly and the needle was left in place for 2 min before it was slowly retracted. Three different cell numbers, 5×10^2^, 3×10^3^, and 1×10^4^, were included in the experiments, and all results were independent of cell number within this range. The mice were examined daily for up to 70 days after tumor cell injection. Moribund mice were killed and autopsied, and the brain was removed for subsequent fluorescence imaging or histological analysis.

### Intra-arterial tumor cell injection

A tuberculin syringe with a 26-gauge needle was used to inject 10^5^ cells suspended in 0.1 ml HBSS into the left ventricle of the heart. A small air-bubble was left in the plunger side of the syringe before injection to facilitate blood entrance. The ribs were visualized by a 1 cm skin incision along the sternum. The needle was inserted vertically into the third intercostal space 1–2 mm to the left of the sternum. Spontaneous and continuous entrance of pulsatile oxygenated blood into the needle hub was used as an indication of proper positioning in the left ventricle of the heart. The cells were injected slowly over a period of ~30 s. The mice were examined daily after tumor cell injection and moribund mice were killed and autopsied. The brain, spinal cord, axial and inguinal lymph nodes, lungs, kidneys, adrenal glands, pancreas, and liver were examined by visual inspection and by fluorescence microscopy.

### Fluorescence imaging

Imaging was performed with an inverted fluorescence microscope equipped with a filter for green light (IX-71; Olympus, Munich, Germany), a black-and-white CCD camera (C4742-95, Hamamatsu Photonics, Hamamatsu, Japan), and appropriate image acquisition software (Wasabi, Hamamatsu Photonics). The fresh brain was imaged immediately after autopsy. The dorsal and ventral surfaces of the brain as well as 3 coronal brain sections with a thickness of approximately 2 mm were imaged at low (x2) and high (x4-x10) magnifications.

### Histological analysis

The brain was fixed in phosphate-buffered 4 % paraformaldehyde. Histological sections were stained with hematoxylin and eosin (HE) by using a standard procedure or immunostained by using a peroxidase-based indirect staining method [30]. An anti-GFP rabbit polyclonal antibody, an anti-CD31 rabbit polyclonal antibody, an anti-MMP-2 rabbit polyclonal antibody, or an anti-MMP-9 rabbit polyclonal antibody (all from Abcam, Cambridge, United Kingdom) was used as primary antibody. Diaminobenzidine was used as chromogen, and hematoxylin was used for counterstaining.

### Quantitative PCR

RNA isolation, cDNA synthesis, and quantitative PCR were performed as described in detail previously for cells in culture [29]. Briefly, gene expression was assessed by using the RT^2^ Profiler PCR Array Human Angiogenesis (PAHS-024A) from SABiosciences (Frederick, MD). Real-time PCR was performed on an ABI 7900HT Fast Real-Time PCR instrument (Applied Biosystems, Carlsbad, CA). Each tumor line was run in three biological replicates. Glyceraldehyde-3-phosphate dehydrogenase (GAPDH) and β-actin (ACTB) were used as normalization genes because these housekeeping genes showed stable expression across the melanoma lines studied here. Thus, each replicate C_T_-value was normalized to the mean C_T_-value of GAPDH and ACTB (ΔC_T_ = C_T_^gene of interest^ – C_T_^mean of GADPH and ACTB^).

### ELISA

Medium samples from cell cultures in exponential growth were collected 24 h after change of medium. Commercial ELISA kits (Quantikine; R&D Systems, Abingdon, United Kingdom) were used according to the manufacturer’s instructions to measure the concentrations of VEGF-A, IL-8, and MMP-2 in the medium samples as described previously [25].

### Statistical analysis

Statistical comparisons of data were performed by one-way analysis of variance followed by the Student-Neuman-Keuls test when the data complied with the conditions of normality and equal variance, and under other conditions by the Kruskal-Wallis one-way analysis of variance on ranks. Statistical comparisons of survival curves were performed using the log-rank test. Probability values of *P* < 0.05 were considered significant. Statistical analysis was performed with SigmaStat statistical software (SPSS, Chicago, IL).

## Results

### Gene expression and secretion of proteins involved in angiogenesis and invasion

**Fig. 1:**
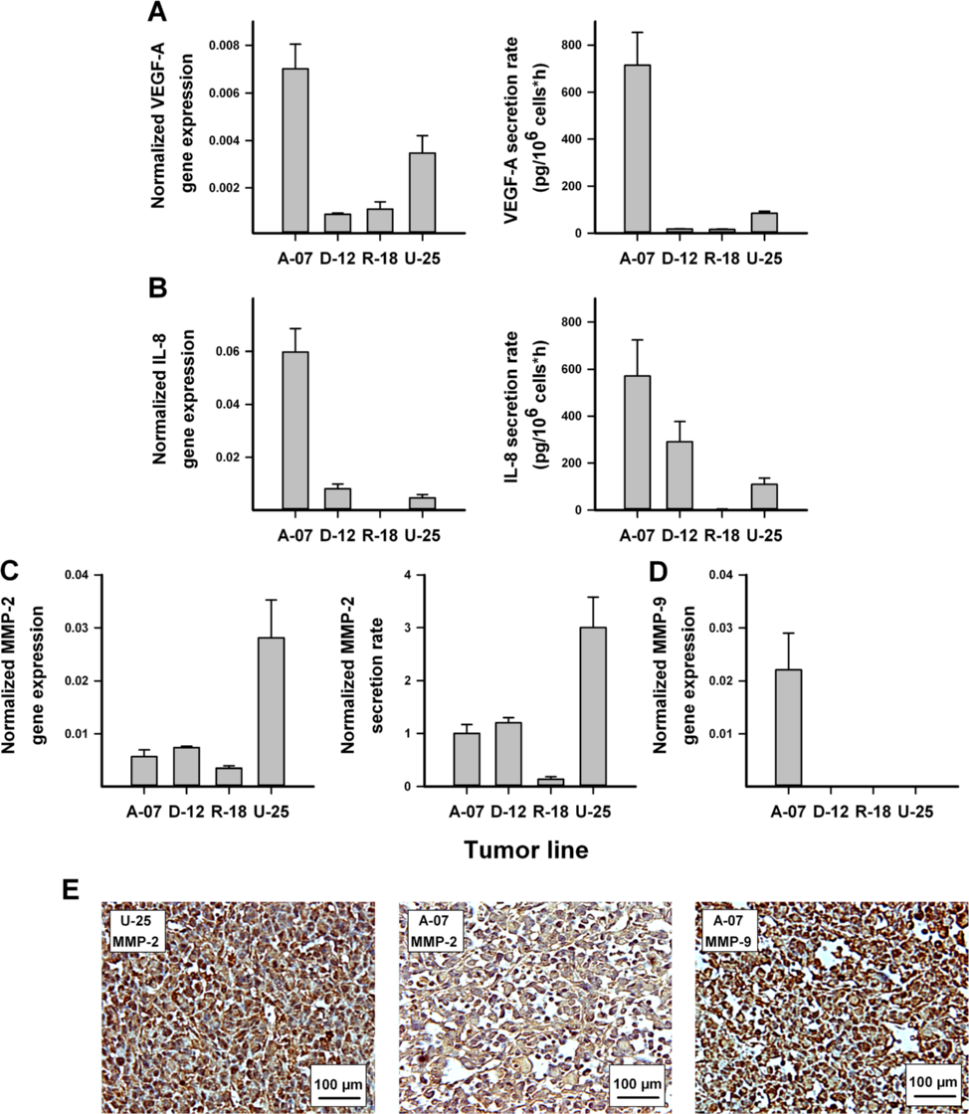
Gene expression and secretion of proteins involved in angiogenesis and invasion. Normalized gene expression and protein secretion rate of VEGF-A (**a**), IL-8 (**b**), and MMP-2 (**c**) and normalized gene expression of MMP-9 (**d**) in the A-07, D-12, R-18, and U-25 lines, and examples of immunohistochemical preparations of the melanoma lines stained for MMP-2 or MMP-9 (**e**). Gene expression was measured with quantitative PCR and was normalized to the mean expression of two housekeeping genes (GAPDH and ACTB). Protein secretion rates were measured with ELISA. Columns and bars represent the mean ± SEM of three (PCR) or five (ELISA) independent experiments

VEGF-A gene expression and secretion rates were significantly higher in the A-07 and U-25 lines than in the D-12 and R-18 lines, and the A-07 line showed 2-fold higher gene expression and 8-fold higher secretion rate than the U-25 line (Fig. 1a; *P* < 0.05). IL-8 gene expression and secretion rates were significantly higher in the A-07, D-12, and U-25 lines than in the R-18 line, and the A-07 line showed 7-fold and 13-fold higher gene expression and 2-fold and 5-fold higher secretion rate than the D-12 and U-25 lines, respectively (Fig. 1b; *P* < 0.05). The U-25 line showed 5-fold, 4-fold, and 8-fold higher MMP-2 gene expression and 3-fold, 2.5-fold, and 22-fold higher MMP-2 secretion rate than the A-07, D-12, and R-18 lines, respectively (Fig. 1c; *P* < 0.05). The A-07 line showed 160-fold and 470-fold higher MMP-9 gene expression than the D-12 and R-18 lines, whereas the U-25 line did not show significant expression of MMP-9 (Fig. 1d; *P* < 0.05). Immunohistochemistry revealed that the U-25 line showed stronger staining for MMP-2 than the A-07, D-12, and R-18 lines, and furthermore, A-07 was the only line that showed detectable staining for MMP-9 (Fig. 1e).

### Intracranial growth pattern following intracerebral injection

**Fig. 2:**
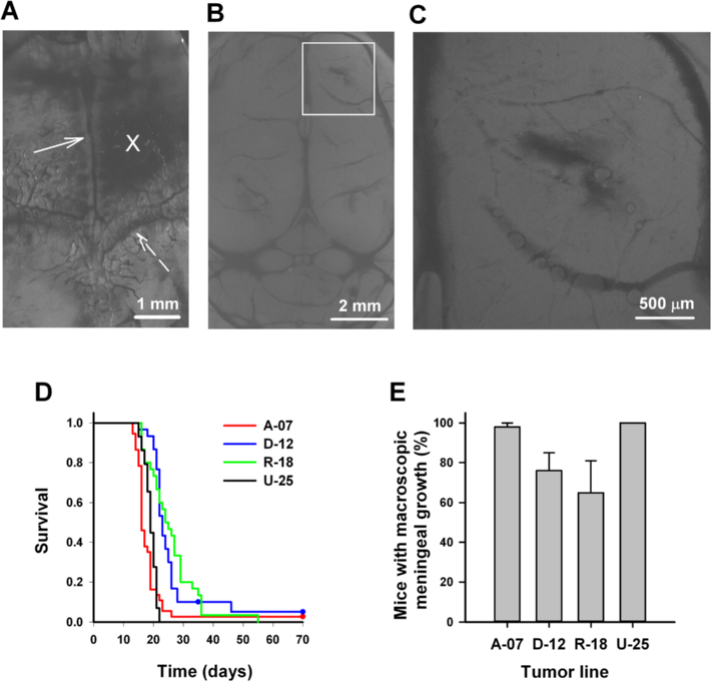
Survival and macroscopic tumor growth following intracerebral injection. **a** Fluorescence image of the dorsal surface of the scull bone showing the injection site (*cross*) located 2 mm anterior to the coronal suture (*dashed arrow*) and 1 mm lateral to the sagittal suture (*solid arrow*). **b**–**c** Low and high magnification fluorescence images of the injection site on the dorsal surface of the brain 1 h after intracerebral injection of 10^4^ GFP-transfected A-07 cells. **d** Survival versus time for mice injected with A-07, D-12, R-18, and U-25 cells. **e** Fraction of mice with macroscopic meningeal tumor growth at autopsy. Columns and bars represent mean and SEM of three separate experiments, each involving ten mice. Survival curves in **d** are based on pooled data from all three experiments and include 30 mice per cell line

Artificial melanoma brain metastases were established by stereotactic injection of A-07, D-12, R-18, and U-25 cells into the right cerebral hemisphere of nude mice (Fig. 2a). The cells were delivered intracerebrally, and no GFP-expressing cells were detected in the leptomeningeal lining of the brain 1 h after injection (Fig. 2b–c). All four lines showed aggressive intracranial growth, and mice injected with A-07 or U-25 cells showed significantly shorter survival than mice injected with D-12 or R-18 cells (Fig. 2d; *P* < 0.0001). Clinical symptoms of intracranial tumor growth differed substantially among the melanoma lines. Mice injected with A-07 cells showed rapid weight loss, loss of general activity, and disconnection of scull sutures due to increased intracranial pressure. Mice injected with U-25 cells showed moderate weight loss, intra- and extracranial bleeding, and visible destruction of scull bone by tumor cells. Approximately 60 % of the mice injected with D-12 cells showed similar symptoms as the U-25 mice, whereas the rest of the D-12 mice developed intracranial bleeding, severe weight loss, or hind leg paralysis. Mice injected with R-18 cells showed gradual weight loss (cachexia) as the only clinical symptom of tumor growth.

At autopsy, macroscopic tumor growth in the brain parenchyma was observed in less than 5 % of the mice, whereas macroscopic tumor growth in the meninges was observed in nearly all mice injected with A-07 or U-25 cells and in a majority of mice injected with D-12 or R-18 cells (Fig. 2e). The clinical symptoms observed in mice injected with A-07 or U-25 cells and a majority of mice injected with D-12 cells could be attributed to meningeal tumor growth, and the meningeal tumors in these mice were large enough to cause visible compression of the right cerebral hemisphere. In contrast, the meningeal tumors of mice injected with R-18 cells grew mainly horizontally, and it was not clear from the macroscopic analysis whether the meningeal tumor growth caused the clinical symptoms observed in these animals.

**Fig. 3:**
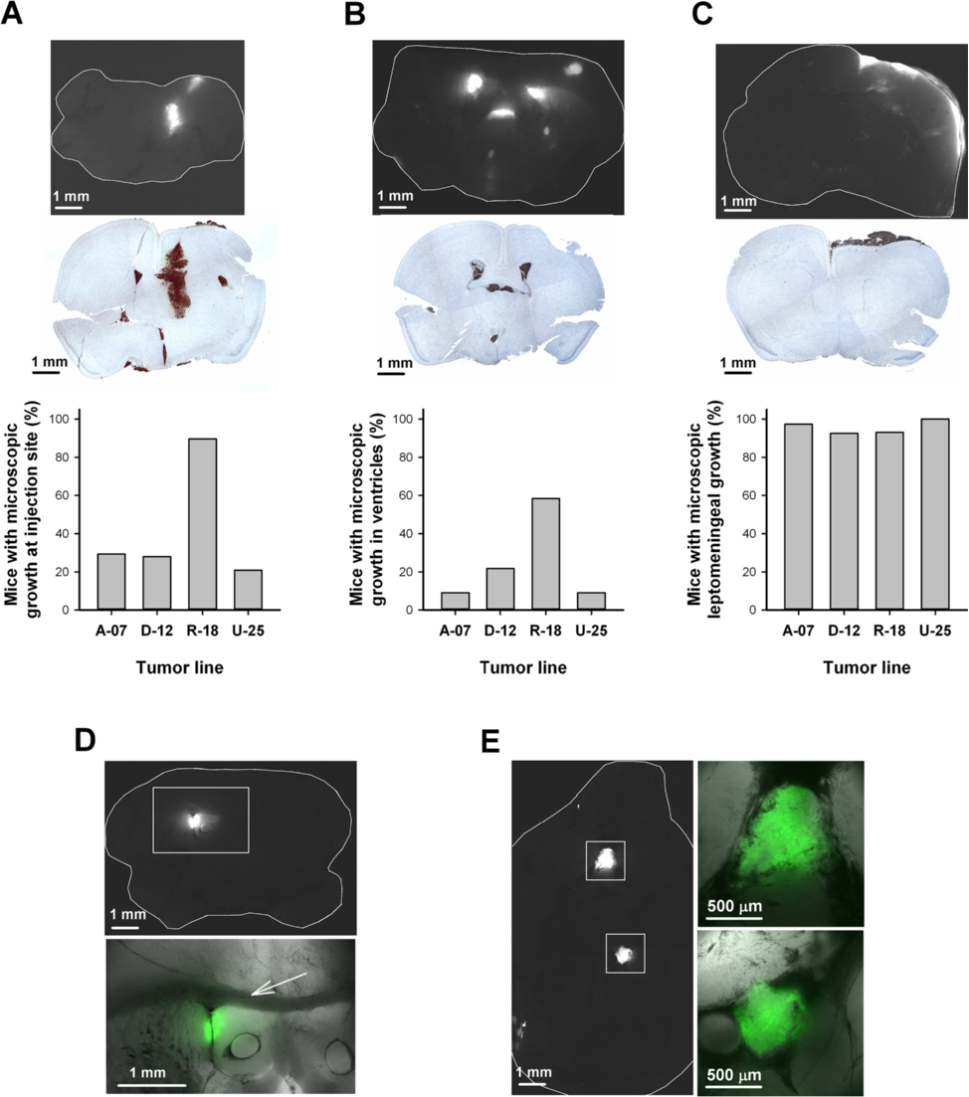
Microscopic growth pattern following intracerebral and intra-arterial injection. **a**–**c** Microscopic tumor growth in the brain after intracerebral injection: representative GFP fluorescence images, histological brain sections stained for GFP, and fraction of mice showing microscopic growth at the injection site (**a**), in the ventricles (**b**), and in the leptomeninges (**c**). **d**–**e** Microscopic tumor growth in the brain after intra-arterial injection: representative fluorescence images showing growth of U-25 cells in the left lateral ventricle (**d**) and on the ventral surface of the brain (**e**). High magnification images (**d**–**e**) are shown as combined transillumination and pseudocolored fluorescence images. Arrow in **d** indicates corpus callosum

To study the microscopic pattern of tumor growth, coronal brain sections were subjected to GFP-fluorescence imaging or GFP immunohistochemistry. Tumor growth was observed in three different locations within the brain: the injection site, the ventricles, and the leptomeninges (Fig. 3a–c). Tumor growth at the injection site and tumor growth in the brain ventricles was observed more frequently in mice injected with R-18 cells than in mice injected with A-07, D-12, or U-25 cells (Fig. 3a and b). Furthermore, microscopic analysis confirmed tumor growth in the leptomeninges of > 90 % of the mice regardless of cell line (Fig. 3c).

### Intracranial growth pattern following intra-arterial injection

To investigate whether the melanoma cells produce a different growth pattern when they reach the brain via the blood stream, the cells were injected intra-arterially into the left ventricle of the heart. All animals became moribund within 3–4 weeks due to extracranial metastases involving the liver, adrenal glands, spinal cord, lungs, and/or lymph nodes, thus limiting the observation time for growth in the brain. Consequently, the brain lesions observed after intra-arterial injection were smaller than the brain lesions observed after intracerebral injection, but large enough to be detected by fluorescence imaging. Similar to the intracerebral injections, the intra-arterial injections produced tumor lesions associated with the brain ventricles (Fig. 3d) and tumor lesions in the leptomeninges (Fig. 3e).

### Intertumor heterogeneity in parenchymal invasion at the injection site

**Fig. 4:**
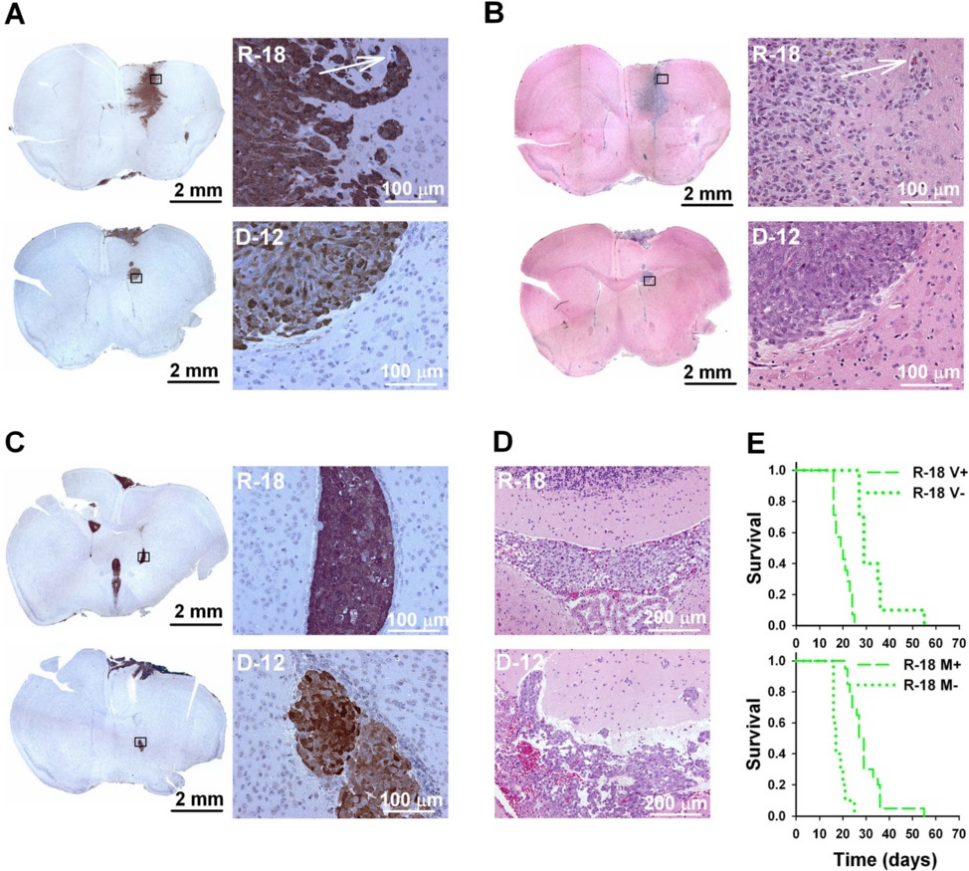
Intertumor heterogeneity in growth and invasion at the injection site and in the ventricles. **a**–**b** Low and high magnification images of GFP-stained (**a**) and HE-stained (**b**) histological brain sections showing tumor growth at the injection site following intracerebral injection of R-18 and D-12 cells. Arrow indicates a blood vessel with co-optive tumor growth. **c**–**d** GFP-stained (**c**) and HE-stained (**d**) brain sections showing tumor growth in the lateral (**c**) and fourth (**d**) ventricles following intracerebral injection of R-18 and D-12 cells. **e** Survival versus time for R-18 mice with and without ventricular tumor growth (V+ and V-), and R-18 mice with and without macroscopic meningeal tumor growth (M+ and M-)

Following intracerebral injection, the R-18 cells were more invasive than the other melanoma lines within the brain parenchyma at the injection site. Thus, R-18 cells showed infiltrative growth with diffuse borders between the tumor and surrounding brain parenchyma, whereas A-07, D-12, and U-25 cells showed well-defined lesions with a distinct tumor border. This is illustrated in Fig. 4a–b showing local tumor growth at the injection site after intracerebral injection of R-18 and D-12 cells. The R-18 cells tended to invade the surrounding brain parenchyma by co-option of blood vessels in the tumor periphery (Fig. 4b). Angiogenic activity at the intracerebral injection site was not detected for any of the melanoma lines.

### Intertumor heterogeneity in ventricular growth and invasion

Ventricular R-18 tumors grew as well-defined lesions within the intraventricular spaces of the brain and showed no signs of invasion into surrounding tissues, whereas invasion from the ventricles and into surrounding tissues was observed in mice injected with A-07, D-12, or U-25 cells. This is illustrated in Fig. 4c–d showing tumor growth in the lateral ventricles (Fig. 4c) and in the fourth ventricle (Fig. 4d) following intracerebral injection of R-18 and D-12 cells.

R-18 mice with ventricular growth (V+) showed significantly shorter survival than R-18 mice without ventricular growth (V-) (Fig. 4e, upper panel; *P* < 0.001). In contrast, R-18 mice with macroscopic meningeal tumor growth (M+) showed significantly longer survival than R-18 mice without macroscopic meningeal tumor growth (M-) (Fig. 4e, lower panel; *P* < 0.001), and this was most likely a result of a lower frequency of ventricular tumor growth in mice with meningeal tumors than in the mice without meningeal tumors (29 % vs 100 %). The survival of R-18 mice after intracerebral injection was therefore limited mainly by ventricular rather than meningeal tumor growth.

### Intertumor heterogeneity in meningeal vascularization and scull bone invasion

**Fig. 5:**
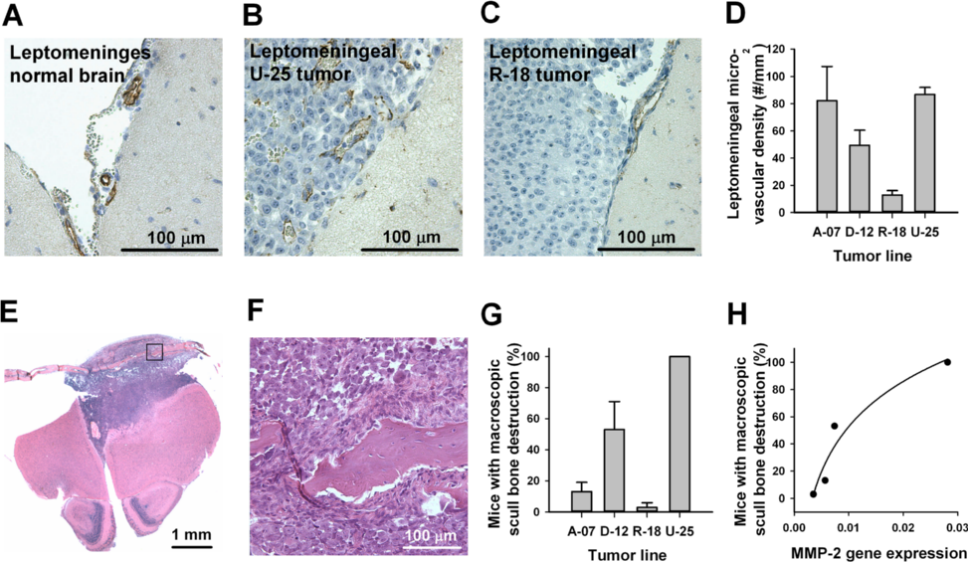
Intertumor heterogeneity in meningeal vascularization and scull bone invasion. **a**–**c** Histological brain sections stained for CD31 to visualize blood vessels in the leptomeninges of a normal brain (**a**), a leptomeningeal U-25 tumor (**b**), and a leptomeningeal R-18 tumor (**c**). **d** Microvascular density in leptomeningeal tumors assessed from CD31 immunohistochemistry. Columns and bars represent mean and SEM of 4 mice. **e** HE-stained coronal section through the brain and overlying scull bone ~1 mm anterior to the site of intracerebral injection of U-25 cells. **f** High magnification image of area indicated in E showing scull bone disrupted by tumor growth. **g** Fraction of mice with macroscopic scull bone destruction at autopsy. Columns and bars represent mean and SEM of three separate experiments, each involving 10 mice. **h** Fraction of mice with macroscopic scull bone destruction versus MMP-2 gene expression assessed by quantitative PCR. Points represent melanoma cell lines. Line represents logarithmic regression curve (y = y_0_ + a · ln x, *R* ^*2*^ = 0.93, *P* < 0.05)

Intracerebral injection produced meningeal A-07, U-25, and D-12 tumors that were highly vascularized, and intra-tumoral bleedings were frequently observed, particularly in U-25 and D-12 mice. Evaluation of CD31-stained histological sections revealed an increased number of blood vessels within leptomeningeal tumors that appeared enlarged and irregular compared to normal leptomeningeal vessels, suggesting angiogenic activity (Fig. 5a–b). In contrast, meningeal R-18 tumors appeared less vascularized, and angiogenic vessels were not detected in CD31-stained histological sections (Fig. 5c). Quantitative analysis showed that the microvascular density was significantly higher in leptomeningeal A-07, D-12, and U-25 tumors than in leptomeningeal R-18 tumors (Fig. 5d; *P* < 0.05).

The melanoma lines showed highly different abilities to invade and disrupt the scull bone. Fig. 5e shows a HE-stained histological section of a meningeal U-25 tumor growing between the brain and the overlying scull bone. The scull bone is in the process of being invaded and disrupted by tumor cells (Fig. 5f). Macroscopic scull bone disruption was observed more frequently in mice injected with U-25 cells than in mice injected with A-07, D-12 or R-18 cells (Fig. 5g; *P* < 0.05). Moreover, the differences among the cell lines in scull bone disruption were associated with differences in expression of MMP-2 (Fig. 5h).

### Melanoma cell invasion along and across the meningeal linings of the brain

**Fig. 6:**
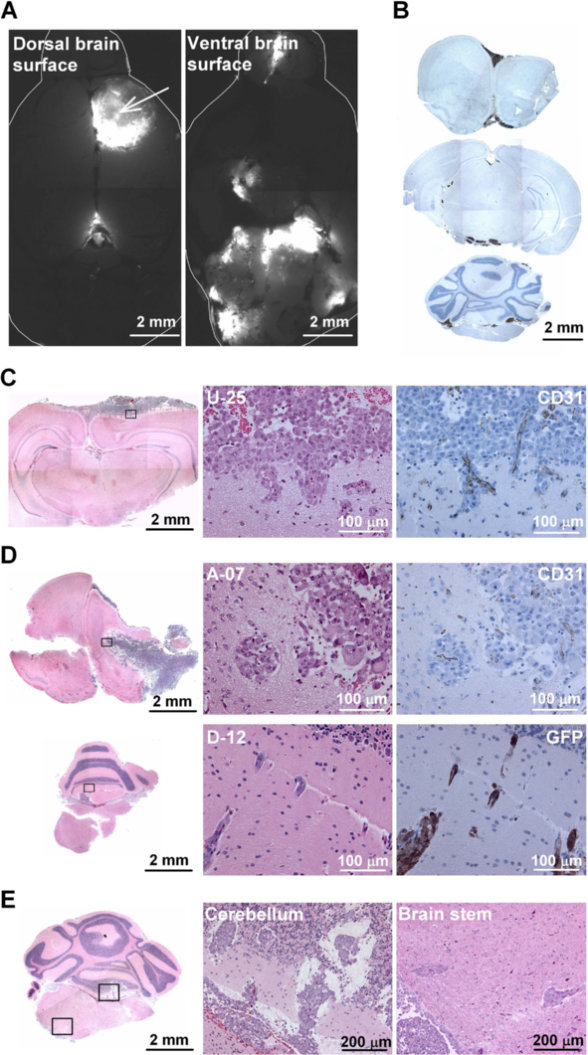
Melanoma cell invasion along and across the meningeal linings of the brain. **a** GFP-fluorescence images of the dorsal and ventral surfaces of the brain after intracerebral injection of D-12 cells. Arrow indicates injection site. **b** GFP-stained histological brain sections after intracerebral injection of R-18 cells. GFP-expressing tumor deposits are present on the dorsal and ventral surfaces of the cerebrum (upper panel) as well as on the ventral surface of the midbrain (middle panel) and cerebellum (lower panel). The three brain sections were obtained ~1 mm anterior, ~3 mm posterior, and ~6 mm posterior to the injection site. **c**–**d** Histological brain sections showing parenchymal invasion of U-25, A-07, and D-12 cells. **e** Histological brain sections showing D-12 cells invading the cerebellum and brain stem after intracerebral injection

All four melanoma lines produced multiple meningeal lesions along the surface of the brain after intracerebral injection, as illustrated in Fig. 6a. GFP immunohistochemistry revealed tumor deposits on the surfaces of multiple brain regions, including the dorsal and ventral surfaces of the cerebrum and the ventral surfaces of the midbrain and cerebellum (Fig. 6b). This growth pattern indicated that the melanoma cells invaded along the meningeal linings of the brain to reach distant parts of the brain surface. In mice injected with A-07, D-12, or U-25 cells, this meningeal invasion was followed by secondary invasion of the brain parenchyma, and two different invasion patterns were observed. Invasion via the perivascular spaces of vessels entering the brain was characteristic of D-12 and U-25 cells (Fig. 6c), and single cell or multicellular migration followed by co-option of microvessels within the brain parenchyma was characteristic of A-07 and D-12 cells (Fig. 6d). Combined invasion along and across the meningeal linings of the brain resulted in multiple brain lesions that involved brain compartments far from the injection site. An example of D-12 cells invading the cerebellum and brain stem is shown in Fig. 6e.

## Discussion

The human melanoma cell lines A-07, D-12, R-18, and U-25 showed aggressive intracranial growth following intracerebral implantation, and the survival of the mice was limited by tumor growth in the meninges and ventricles rather than by local tumor growth at the injection site. A similar growth pattern was observed after intra-arterial injection, suggesting that a preference for growth in the meninges and ventricles was a characteristic feature of the melanoma cells. All four cell lines are established from subcutaneous metastases [24], and the results are thus in accordance with previous studies showing that human melanoma cell lines derived from non-CNS metastases produce lesions mainly in the meninges and ventricles after intracarotid artery injection, whereas cell lines derived from brain metastases produce lesion mainly in the brain parenchyma [16, 17]. In the present study, the melanoma cells induced angiogenesis in the meninges, whereas angiogenic activity was not detected within the brain parenchyma. Blood vessels in the brain parenchyma form a tight blood brain barrier, whereas meningeal capillaries are lined by fenestrated endothelium and are thus more similar to capillaries found in most extracranial organs [16, 17]. The melanoma cells’ preference for growth in the meninges may therefore reflect differences between meningeal and parenchymal blood vessels in their susceptibility to angiogenic factors secreted by the melanoma cells.

The aggressiveness of meningeal tumors differed substantially among the melanoma lines, and these differences were associated with differences in angiogenic activity and in expression of VEGF-A and IL-8. Thus, A-07, D-12, and U-25 cells grew more aggressively in the meninges, showed higher microvascular density in leptomeningeal tumors, and showed higher expression and secretion of VEGF-A and IL-8 than R-18 cells. In contrast, all four melanoma lines appeared to rely primarily on vascular co-option for growth and invasion of the brain parenchyma regardless of VEGF-A expression. Our data thus suggest that also melanoma cells with high expression of VEGF-A may show a co-optive and infiltrative growth pattern within the brain. The observation time for tumor growth in the brain parenchyma was limited by the aggressiveness of the meningeal tumors and, therefore, it is possible that also parenchymal lesions induce angiogenesis in a later growth stage. For example, parenchymal lesions may develop regions with hypoxic tissue during expansion, and changes in the extent of hypoxia during growth may cause dynamic changes in the expression of angiogenic factors influencing the form of vascularization, leading to vascularization by angiogenesis in addition to co-option in large lesions.

The correlations reported here suggest that VEGF-A and IL-8 are required for the growth of brain metastases in our artificial metastasis models. This suggestion is in accordance with an earlier work with the same melanoma lines showing that treatment of tumor-bearing mice with neutralizing antibody against VEGF-A or IL-8 decreased the microvascular density and the growth rate of the primary tumor and reduced the incidence of spontaneous metastases, including lymph node, pulmonary, hepatic, and brain metastases [26]. Our suggestion is also consistent with a study by Yano et al. [22], reporting that VEGF-A expression is necessary but not sufficient for production and growth of brain metastases.

The melanoma lines showed highly different invasion patterns, and differences in invasiveness was associated with differences in expression of MMP-2 and MMP-9. Thus, R-18 cells invaded locally both within the brain parenchyma at the injection site and within the leptomeninges and ventricles, but showed low ability to invade across the barriers separating different brain compartments. In contrast, A-07, D-12, and U-25 cells frequently invaded the brain parenchyma from the meninges or ventricles. The brain parenchyma and the pia mater are separated by the glia limitan, consisting of astrocyte foot processes attached to a parenchymal basal lamina [31]. MMP-2 and MMP-9 have previously been shown to facilitate the migration of leukocytes across the glia limitans [31, 32]. In this study, we found higher expression of MMP-2 in the A-07, D-12, and U-25 lines than in the R-18 line, and higher expression of MMP-9 in the A-07 line than in the other lines. Lower expression of these matrix metalloproteinases could thus potentially explain the low invasiveness of R-18 cells across the glia limitan.

A-07, D-12, R-18, and U-25 cells growing in the meninges also differed considerably in their ability to invade and destruct the overlying scull bone. These differences were not directly related to angiogenic activity in the meninges. Thus, although A-07 and U-25 mice showed similar aggressiveness and angiogenic activity in the meninges, the ability to invade and destruct the scull bone was substantially higher for U-25 cells than for A-07 cells. Interestingly, the fraction of mice with macroscopic scull bone destruction was closely associated with the expression of MMP-2. Overexpression of MMP-2 has previously been shown to promote both brain and bone metastasis in a preclinical model of breast cancer and to promote bone absorption in a prostate cancer model [33, 34]. Our data suggest that MMP-2 may be associated with bone invasion also in melanoma, and this warrants further investigations.

In a previous study, we revealed that A-07, D-12, and T-22 melanoma cells cultured at acidic extracellular pH show increased secretion of MMP-2 and MMP-9, enhanced invasiveness in vitro, and enhanced potential to develop experimental pulmonary metastases in BALB/c *nu/nu* mice [27]. Moreover, acidity-induced experimental pulmonary metastasis was inhibited by treatment with the general MMP inhibitor GM6001 and the general cysteine proteinase inhibitor E-64 [27], suggesting that MMP-2 and MMP-9 are required for the development of pulmonary metastases in our melanoma models. Because brain metastases frequently originate from pulmonary metastases, it is likely that MMP-2 and MMP-9 are required for the development of spontaneous brain metastases also. Other members of the MMP family may also be involved in melanoma brain metastasis; however, we have no evidence from our models that MMP-1, MMP-13, or MT1-MMP is required for metastatic growth in the brain.

Melanomas have a high tendency to produce multiple brain lesions, and Kienast et al. have suggested that this tendency is the result of a high motility of melanoma cells within the brain [15]. Our data are consistent with this suggestion, showing that melanoma cells injected into a specific location in the right cerebral hemisphere are able to produce multiple brain lesions involving brain compartments far from the injection site. Moreover, our data show that the melanoma cells used the meningeal surfaces of the brain as transport routes. Leptomeningeal involvement has been shown to be correlated with an increased number of parenchymal lesions in a large retrospective study of patients with stage IV melanoma [8]. Furthermore, autopsy studies have suggested that the true incidence of leptomeningeal metastasis may be substantially higher than the clinical incidence [2, 3]. Consequently, efficient treatment strategies for patients with melanoma brain metastases may need to target melanoma cells in the meninges as well as in the brain parenchyma.

New treatment strategies for patients with melanoma brain metastases are highly needed, and antiangiogenic therapy targeting the VEGF-A pathway has been suggested as a potential strategy [35]. However, patients who harbor brain metastases with a co-optive growth pattern will most likely not respond to antiangiogenic agents. Although VEGF-A-dependent angiogenesis is a characteristic feature of malignant melanoma [36], anti-VEGF-A therapy may have limited potential in patients with melanoma brain metastases due to a tendency of melanoma cells to grow by vascular co-option in the brain despite high expression of VEGF-A. Metastases with a co-optive growth pattern are also more difficult to treat with surgical resection or stereotactic radiosurgery due to the diffuse borders between tumor and normal tissue.

## Conclusions

The human melanoma cell lines A-07, D-12, R-18, and U-25 showed characteristic patterns of vascularization and invasion in the brain, and these differences were associated with differences in expression of the angiogenic factors VEGF-A and IL-8 and the matrix metalloproteinases MMP-2 and MMP-9. Furthermore, the melanoma cells showed a co-optive and infiltrative growth pattern within the brain parenchyma, and produced multiple brain lesions after intracerebral injection by using the meningeal linings of the brain as a route of transport. Our study suggests that melanoma brain metastases may grow by vascular co-option in the brain despite high expression of VEGF-A and, moreover, it suggests that efficient treatment strategies for patients with melanoma brain metastases may need to target melanoma cells within the meninges as well as the brain parenchyma.
